# Generation and Characterization of a Spike Glycoprotein Domain A-Specific Neutralizing Single-Chain Variable Fragment against Porcine Epidemic Diarrhea Virus

**DOI:** 10.3390/vaccines9080833

**Published:** 2021-07-29

**Authors:** Chia-Yu Chang, Yong-Sheng Wang, Jou-Fei Wu, Tzu-Jing Yang, Yen-Chen Chang, Chanhee Chae, Hui-Wen Chang, Shang-Te Danny Hsu

**Affiliations:** 1 Institute of Biological Chemistry, Academia Sinica, Taipei 11529, Taiwan; flywinds11@gmail.com (C.-Y.C.); willywang13@gate.sinica.edu.tw (Y.-S.W.); jing0526@gate.sinica.edu.tw (T.-J.Y.); 2 Institute of Biochemical Sciences, National Taiwan University, Taipei 10617, Taiwan; 3 Graduate Institute of Molecular and Comparative Pathobiology, School of Veterinary Medicine, National Taiwan University, Taipei 10617, Taiwan; a10153018@gmail.com (J.-F.W.); chyenjean@gmail.com (Y.-C.C.); huiwenchang@ntu.edu.tw (H.-W.C.); 4 Department of Veterinary Pathology, College of Veterinary Medicine, Seoul National University, Seoul 151-742, Korea; swine@snu.ac.kr

**Keywords:** porcine epidemic diarrhea virus, single-chain variable fragment (scFv), neutralizing antibody

## Abstract

The emergence of the genotype (G) 2 and re-emergence of the G1 porcine epidemic diarrhea virus (PEDV) has caused severe economic impacts in the past decade. Developments of efficient vaccines against new variants of PEDV have been challenging, not least because of the difficulties in eliciting mucosal and lactogenic immunity. A single-chain fragment variable (scFv) capable of efficient antigen recognition is an alternative to vaccination and treatment of a viral infection. In the present study, the variable regions of the light chain and the heavy chain of a G2b PEDV spike domain A (S1^A^)-specific neutralizing monoclonal antibody (mAb) were sequenced, constructed with a (G4S) x3 linker, and produced by a mammalian protein expression system. Our results demonstrated that the PEDV S1^A^ domain scFv was able to bind to S proteins of both G1 and G2b PEDVs. Nevertheless, the scFv was only capable of neutralizing the homologous G2b PEDV but not the G1 PEDV. The binding ability of the G2b-specific neutralizing scFv was not able to predict the neutralizing ability toward heterologous PEDV. The anti-PEDV S1^A^ scFv presented herein serves as a potential therapeutic candidate against the virulent G2b PEDV.

## 1. Introduction

Porcine epidemic diarrhea virus (PEDV) is a contagious swine enteric virus that causes porcine epidemic diarrhea (PED) in neonatal and suckling piglets, which impacts the swine industry all over the world [1,2]. The historic genotype (G)1 PEDV was first identified in the late 1970s in Belgium and subsequently became an endemic disease over the past decades in Europe and Asia [3,4,5] with sporadic outbreaks [4]. A new variant of PEDV, which belongs to G2 based on the phylogenetic analysis, has emerged since 2010 in China and rapidly spread across North America and Asia [6,7,8,9]. This new PEDV variant caused high morbidity and high mortality in neonatal piglets [4,10,11]. Effective vaccination is a highly sought-after solution to mitigate a PEDV-associated endemic. An ideal PED vaccine should be capable of inducing passive lactogenic immunity from sows to protect neonatal piglets [12,13]. A number of vaccines against G2 PEDV are conditionally licensed in the US and Korea to mitigate the outbreaks, albeit with controversies about the efficacy of the vaccines [14,15]. Importantly, there is hitherto no effective prophylactic method and therapeutic tool to be used in the field to control seasonal outbreaks of PED in neonatal piglets.

PEDV is a single positive-stranded RNA virus that belongs to the genera *Alphacoronavirus* in the family *Coronaviridae* [1]. The genome size of PEDV is approximately 28 kilo-base (kb) to encode for 4 structural proteins, namely spike (S, 180–220 kDa), nucleocapsid (N, 55–58 kDa), membrane (M, 27–32 kDa), and envelope (E, 7 kDa), and 17 non-structural proteins (nsp1-nsp16, and ORF3) [1]. The S protein modulates host recognition, receptor binding, membrane fusion, and harbors neutralizing epitopes. It is, therefore, a major target for the development of vaccines and therapeutics. The S protein of PEDV (PEDV-PT strain, GenBank: KY929405.1) is 1383 amino acids (a.a.) in length, encompassing six functional domains, namely S1^0^ (residues 1–219), S1^A^ (residues 219–509), S1^B^ (residues 509–639), S1^CD^ (residues 639–729), S2 (residues 730–1334), and the terminal transmembrane domain [16,17,18]. Several neutralizing epitopes of historic PEDV (G1) have been identified, including collagenase-26K equivalent (COE, residues 501–640) [19], S1D (residues 640–794) [20], and 2C10 (residues 1373–1379) [20]. Meanwhile, the S1^0^, S1^A^, S1^B^, and the N-terminus of S2 were proposed to have neutralizing epitopes of the new variants of PEDV (G2) [17,18,21]. 

Understanding the structural and functional characteristics of epitopes is essential for immunological therapeutics developments. In human medicine, therapeutic antibodies have been developed since the 1980s. They have become an integral part of modern medicine in treating many diseases, including infectious, oncogenic, metabolic, autoimmune diseases, and even aging-associated disorders [22,23,24]. Several therapeutic antibodies have been deployed successfully and have been commercialized to treat viral and bacterial infections in humans [23]. Along with the increasing need in the market, developments of therapeutic antibodies are being made to tackle severe infectious human diseases such as dengue fever [25], influenza [26], Ebola [27], and COVID-19 [28]. Efforts to develop therapeutic antibodies against animal diseases are also actively being made in veterinary medicine [29,30]. However, success has been limited in controlling swine diseases, which is one of the most valuable agricultural industries worldwide.

We have previously reported a novel neutralizing monoclonal antibody (NmAb), E10E-1-10, that targets a conformational epitope within the S1^A^ domain of S protein of PEDV [18]. In the present study, we determined the DNA sequence encoding for the variable regions of E10E-1-10 by using template-switch specialized reverse transcription-PCR (RT-PCR) [31,32]. An scFv corresponding to E10E-1-10 was constructed and expressed in a mammalian protein expression system, the HEK293 Expi cell that can be cultured in suspension in large volumes, followed by immobilized metal affinity chromatography (IMAC), and size-exclusion chromatography (SEC) to yield highly purified recombinant scFv in milligram quantities. The biological function of recombinant scFv was evaluated by immunostaining, neutralization of viral infection, enzyme-linked immunosorbent assays (ELISA), and immunoprecipitation assays. The results provided good evidence to support the potential use of the scFv for diagnostic and therapeutic purposes against PEDV.

## 2. Materials and Methods

### 2.1. RNA Extraction and Reverse Transcription of the Variable Regions of PEDV S1^A^-Specific mAb

The messenger ribonucleic acid (mRNA) encoding for the variable regions of E10E-1-10 was sequenced by using the template-switch specialized reverse transcription-PCR (RT-PCR) [32]. Briefly, total mRNA was extracted from the freshly harvested hybridoma cells by using the RNeasy Mini Kit (Qiagen, Hilden, Germany) following the manufacturer’s instructions. To synthesize sequence-labeled cDNA, SMARTScribe Reverse Transcriptase kit (Clontech, Mountain View, CA, USA), custom-sequence template-switch oligonucleotide (TSO), and sequence-specific primers for the kappa chain (mIgK RT), lambda chain (mIgL RT), and heavy chain (mIgHG RT) of murine IgG were used (Table 1). For each reaction, 100 ng mRNA, 1 μL of 10 mM primer (mIgK RT, mIgL RT, or mIgHG RT), and 1 μL of 10 mM dNTP were mixed and incubated at 72 °C for 3 min. Subsequently, 2 μL of 5x SMARTScribe buffer (Clontech), 1 μL of 20 mM DTT (Clontech), 3 μL of 10 μM TSO, 0.25 μL of RNAase inhibitor (Roche, Basel, Switzerland), and 0.5 μL of 100 U/μL SMARTScribe Reverse Transcriptase (Clontech) were added to the mixture and the reaction was filled to 20 μL with RNAse-free water. The mixture was incubated at 42 °C for 60 min following a 5-min incubation at 70 °C. The cDNA was stored at −20 °C until use.

### 2.2. Touchdown PCR for Amplifying the Variable Regions of PEDV S1^A^-Specific NmAb 

To obtain the sequences of the variable region of the mAb, the universal custom forward primer, ISPCR, together with chain-specific reverse primers (mIgKpcr, mIgLpcr, or mIgHGpcr) were used to amplify the sequences of the corresponding chain as described previously [32] (Table 2). For each reaction, 2 μL of cDNA, 10 μL of 2x AmaR One PCR mixture (GeneDireX, Taichung City, Taiwan), 1 μL of 10 mM universal forward primer, 1 μL of 10 mM chain-specific reverse primer, and 6 μL of PCR-grade water were prepared. The condition of the thermal cycler was defined as follows: 94 °C for 3 min, 12 cycles of 95 °C for 30 s, 60 °C for 30 s (−0.5 °C per cycle), and 72 °C for 1 min; and directly followed by 18 cycles of 95 °C for 30 s, 56 °C for 30 s, and 72 °C for 1 min. The final extension was 5 min at 72 °C. The expected sizes of amplicon were all approximately 550–600 bp. The sequences were analyzed by the IgBlast tool of NCBI to verify the framework regions (FRs) and the complementarity determining regions (CDRs) of each chain.

### 2.3. Construction and Expression of the Single-Chain Variable Fragment (scFv)

The sequences encoding for the light-chain variable region (VL) and heavy-chain variable region (VH) were linked by a (G_4_S)_3_ linker, one of the most commonly used linkers in the construction of scFv [33]. The tissue plasminogen activator (tPA) signal sequence was introduced at the 5′ end of the coding sequence to help the secretory process. The DNA sequence was synthesized (GeneScript, Piscataway, NJ, USA) and cloned into the pcDNA™3.1/V5-His TOPO vector by using *BamHI* and *NotI* restriction enzymes. After ligation, the plasmid was amplified by the One Shot™ TOP10 competent *E. coli* (Thermo, Waltham, MA, USA) and extracted by the EasyPrep EndoFree Maxi Plasmid Extraction Kit (TOOLs, New Taipei City, Taiwan). To transfect 1 L of Expi293F™ cells at a concentration of 3 × 10^6^ cells/mL, 2.7 mL of ExpiFectamine™ 293 transfection reagent (Thermo) were pre-diluted in 50 mL of Opti-MEM (Gibco, Invitrogen, Waltham, MA, USA) and mixed with 1 mg of the DNA plasmid pre-diluted in 50 mL of Opti-MEM (Gibco, Invitrogen). The mixture was incubated at room temperature for 20 min prior to transfection. After adding the transfection mixture, the Expi293F™ cells were cultured at 37 °C, supplemented with 8% CO_2_ with constant shaking at 125 rpm. Twenty hours after transfection, 5 mL of ExpiFectamine^TM^ 293 transfection enhancer 1 (Gibco, Invitrogen) and 50 mL transfection enhancer 2 (Gibco, Invitrogen) were added to boost recombinant protein expression for a total of four days. At the end of the four-day transient expression, the supernatant and cell lysates of Expi293F™ cells were collected for SDS-PAGE and Western blotting analyses to confirm the expression level of the target scFv.

### 2.4. Purification of scFv by Using Immobilized Metal Affinity Chromatography (IMAC) and Size Exclusion Chromatography (SEC)

After filtration by using a 0.22 μm filter cup (Thermo), the supernatant was mixed with the binding buffer (500 mM Tris-HCl (Sigma, Aldrich, St. Louis, MO, USA), 1.5 M sodium chloride (Sigma), and 50 mM imidazole (Sigma), pH 7.8) with a ratio of 10:1. The HisPur™ Cobalt Resin (Thermo), which was pre-washed with sterile water and TBA buffer (50 mM Tris-HCl (Sigma), 150 mM sodium chloride (Sigma), 0.02% sodium azide (Sigma), pH 7.6) was added to the supernatant in the binding buffer in a ratio of 1:100. After overnight incubation at 4 °C with regular stirring, the cobalt resin was collected by the Glass Econo-Column^®^ Column (Bio-Rad, Hercules, CA, USA), and washed with the 5-fold resin volume of wash buffer (20 mM Tris-HCl (Sigma), 300 mM sodium chloride (Sigma), and 10 mM imidazole (Sigma), pH 7.6). The target protein was eluted by using the elution buffer (20 mM Tris-HCl (Sigma), 300 mM sodium chloride (Sigma), and 150 mM imidazole (Sigma), pH 7.8). The eluent was concentrated to 0.5 mL for further purification in PBS (Sigma) by using a Superdex™ 75 Increase column (GE Healthcare, Chicago, IL, USA) coupled to an AKTA UPC10 FPLC system (GE Healthcare). 

### 2.5. Validation of the scFv by Immunocytochemical Staining (ICC) and Immunofluorescence Assay (IFA) 

Vero cells were seeded on 96-well plates (Thermo) one day before the challenge. The cells were washed with Dulbecco’s phosphate-buffered saline (dPBS, Gibco, Invitrogen) and inoculated with 100 μL of 500 TCID_50_/mL of PEDV-PT passage 5 (G2b PEDV) or PEDV-CV777 (G1 PEDV) diluted in the TPA medium, a DMEM-based medium (Gibco, Invitrogen) supplemented with 0.3% tryptose phosphate broth (Sigma), 0.02% yeast extract (Acumedia, Lansing, CA, USA), and 10 μg/mL trypsin (Gibco, Invitrogen). After a visible cytopathic effect (CPE), the cells were fixed with 80% acetone for 20 min and air-dried for another 30 min. After washing with PBS three times, the purified scFv was diluted to 5 μg/mL with PBS and applied to the wells for 1 h of incubation. To verify the signals, a blank filled with PBS was included as a background control. Following three washing steps with PBS, the 1000× diluted anti-V5 antibody (Invitrogen) was used for another hour of incubation to probe the V5 tag on our recombinant scFv. The goat-anti-mouse IgG conjugated with HRP (Dako, Santa Clara, CA, USA) or conjugated with FITC (Jackson Laboratory, Bar Harbor, ME, USA) were utilized as the secondary antibody and was incubated with the plates for 1 h. Sequentially, the EnVision-DAB+ system (Dako) was used to visualize the signals of HRP conjugated secondary antibody. On the other hand, a ZOE™ Fluorescent Cell Imager (Bio-Rad) was operated to observe the signals of FITC conjugated secondary antibody and also DAPI mounting solution. 

### 2.6. Binding Ability Estimation of scFv toward Homogenous and Heterogeneous PEDV Virions by Indirect ELISAs

The purified virions of PEDV-PT and PEDV-CV777 were diluted to 2 μg/mL with coating buffer (KPL, SeraCare, Milford, MA, USA) and respectively coated onto the Nunc maxisoap strips (Thermo) at 4 °C overnight. The strips were washed with 200 μL of washing buffer (KPL, SeraCare) six times and sequentially blocked with 300 μL of blocking buffer (KPL, SeraCare) for 1 h. The recombinant scFv were serially two-fold diluted from 20 μg/mL to 1.25 μg/mL, and applied on the strips under the condition of 100 μL/well, and incubated for 1 h at room temperature. After washing with 200 μL of washing buffer (KPL, SeraCare) six times, the 1000× diluted anti-V5 antibody (Invitrogen) was incubated with the strips for another hour to probe the V5 tag on the scFv. Following the six washing steps as mentioned above, the 1000× diluted goat-anti-mouse IgG HRP (KPL, SeraCare) was incubated with the strips for 1 h. Fifty microliters of ABTS^®^ Peroxidase Substrate (KPL, SeraCare) were added after the strips were completely washed, and the coloration step was stopped by providing 50 μL of stopping solution (KPL, SeraCare). The signals were detected at 405 nm by using the EMax Plus Microplate Reader (Molecular Devices, San Jose, CA, USA). 

### 2.7. Binding Ability of scFv with Purified PEDV S Protein by Using Immunoprecipitation Assay 

The ectodomain of the S protein of PEDV (GeneBank no. HC070225-S), hereafter PEDV S protein, was constructed, expressed in Expi293F™ cells in a secreted form, and purified from the culture medium as described previously [34]. To conduct the immunoprecipitation pull-down assay, 25 pmol of PEDV S protein (700 kDa in molecular weight as a homotrimer) was mixed with 125 pmol of scFv (30 kDa in molecular weight) and incubated at 37 °C for 3 h with regular shaking. A negative control of scFv only without the PEDV S protein was included. After incubation, the mixture was filtrated by a 100 kDa molecular weight cut-off (MWCO) spin column (Millipore, MA, USA) with centrifugation under 11,000× *g* for 5 min. The flow-through was discarded, and the column was washed with 10 column volumes (400 µL/per wash, 10 washes) of dPBS to remove the unconjugated scFv. The mixture was concentrated to 100 μL after washing and analyzed by SDS-PAGE. Then, 13 µL of the concentrated mixture were mixed with 2 μL of 10× NuPAGE Reducing agent (Invitrogen) and 5 μL of 5× NuPAGE Sampling buffer (Invitrogen), followed by thermal denaturation at 95 °C for 5 min before applying to the 10% SDS-PAGE and stained with Coomassie Brilliant Blue R250 (CBR-250, Thermo). 

### 2.8. Estimation of Binding Ability between scFv and PEDV S Protein by SEC

The binding ability of the scFv toward the PEDV S protein was estimated by SEC. First, 380 pmol of PEDV S protein was mixed with 830 pmol scFv and incubated at room temperature for 1 h. The mixture was filtered by a 0.22 μm spin column (Millipore) before being separated by a Superose 6 10/300 GL column (GE Healthcare) in TBA buffer coupled to an AKTA UPC10 FPLC System (GE Healthcare). The protein sample was monitored by the UV absorbance at 280 nm (UV_280_) and fractionated with 0.5 mL per fraction. The fractions that should signify UV_280_ absorbance were analyzed by Western blotting. The proteins were denatured by adding 2 μL of 10× NuPAGE Reducing agent (Invitrogen), 5 μL of 5× NuPAGE Sampling buffer (Invitrogen), and boiled at 95 °C for 5 min. The protein samples were separated by a 10% SDS-PAGE separating gel and transferred to a polyvinylidene difluoride (PVDF) membrane (Bio-Rad). The membrane was blocked with 5% skim milk for 30 min, and probed with 1:5000 diluted anti-V5 tag antibody (Invitrogen) for 1 h at room temperature. After thorough washing, the 1:10,000 diluted anti-mouse IgG antibody with HRP conjugation (Jackson ImmunoResearch Laboratories) was added. After 1 h of incubation and adequate washing, the V5-tag-positive protein signals were detected by using ClarityTM Western ECL Blotting Substrate (Bio-Rad) and visualized by a ChemiDoc XRS+ Imaging System (Bio-Rad).

### 2.9. Neutralizing Test 

Vero cells were maintained in the DMEM (Gibco, Invitrogen) supplied with 10% FBS (Gibco, Invitrogen) and antibiotic-antimycotic (Gibco, Invitrogen) and were seeded on 96-well cell culture plates (Thermo) to reach 90% confluency on the following day. The purified scFv was two-fold serially diluted from 25 μg/mL to 0.78 μg/mL in the TPA medium and mixed with constant 200 TCID_50_/mL PEDV-PT-passage 5 or PEDV CV777 strain. The controls without adding scFv (the diluted virus only) and without treatment (normal cells fed in TPA medium) were also included in both assays. The virus-antibody mixtures were incubated at 37 °C for 2 h. The Vero cells were gently washed with 200 μL of TPA medium twice, and the virus-scFv mixtures or the controls were inoculated onto the cells in each well, respectively. The cytopathic effect (CPE) of PEDV, which was expected as syncytial cells, was examined at 24, 48, and 72 h after inoculation. 

## 3. Results

### 3.1. Sequencing of the Variable Regions of Neutralizing Monoclonal Antibody

**Figure 1:**
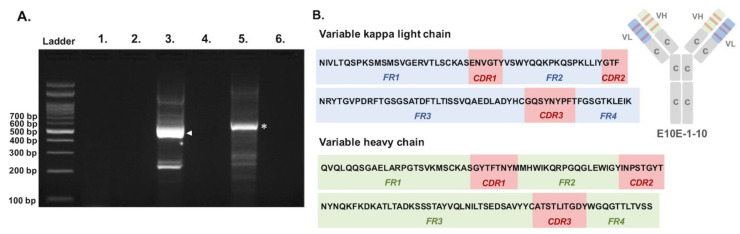
Sequencing of the variable regions of PEDV neutralizing monoclonal antibody, E10E-1-10. (**A**) The PCR reactions were conducted by using one general forward primer and three specific reverse primers, which target on variable lambda light chain (Lanes 1 and 2), variable kappa light chain (Lanes 3 and 4), and variable heavy chain (Lanes 5 and 6). Lanes 2, 4, and 6 were negative controls for each PCR reaction. Major bands sized about 500–600 bp were obtained from Lane 3 (the target amplicon of variable kappa light chain was labeled with arrowhead) and Lane 5 (the target amplicon of variable heavy chain was labeled with asterisk) and sent for sequencing. (**B**) Deduced amino acid sequences and the predicted FRs and CDRs of the variable kappa light chain and variable heavy chain.

The complementary sequences of the variable regions of the heavy and light chains were successfully amplified by using three pairs of general primers. Two amplicons sized approximately 500–600 bp were obtained from PCRs targeting variable kappa light chain and variable heavy chain, respectively (Figure 1A), thus enabling the deduction of the protein sequences corresponding to the variable regions of E10E-1-10, a PEDV-PT-specific NmAb (Figure 1B). The framework regions (FRs) and complementary determining regions (CDRs) of the variable heavy chain and variable light chain, which belong to the kappa subtype, were analyzed and predicted by the IgBlast tool of NCBI. 

### 3.2. Construction, Expression, and Purification of scFv from Expi293F™ Cells

**Figure 2:**
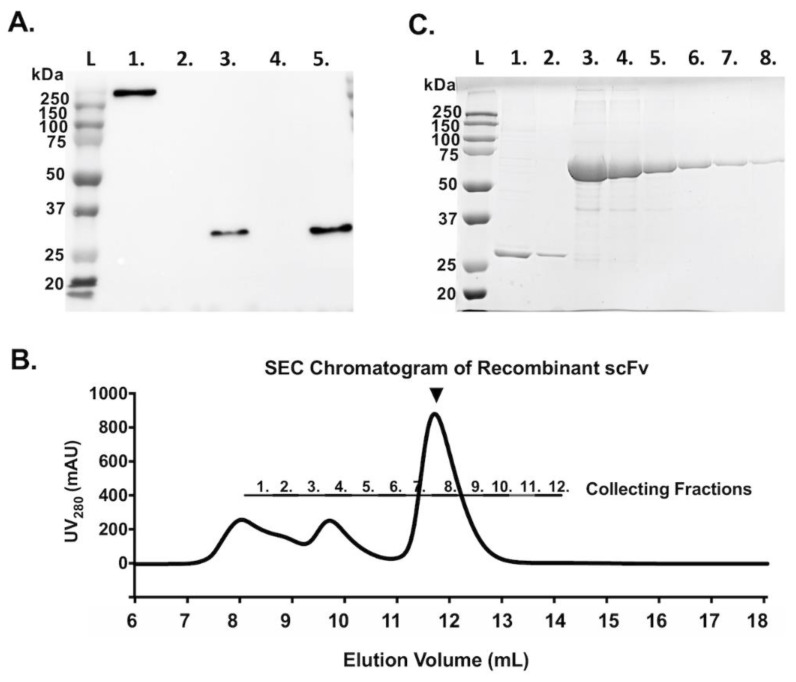
Production of recombinant scFv. (**A**) The expression of recombinant scFv in Expi293F™ cells was confirmed by Western blotting. The expected size of the recombinant scFv is approximately 30 kDa. Lane 1: The His-tagged PEDV S protein was used as a positive control. Lane 2: The cell lysates from non-transfected HEK 293 cells. Lane 3: The cell lysates from scFv-transfected cells. Lane 4: The supernatant from non-transfected cells. Lane 5: The supernatant from scFv-transfected Expi293F™ cells. L: Protein ladder. (**B**) Size-exclusion chromatogram of the recombinant scFv purified by IMAC. UV280 detected the protein content in mAU. The black arrowhead indicates the expected major peak of the scFv. (**C**) The expression and purity of the purified scFv in Expi293F™ cells. Lane 1: two-fold dilution of the purified scFv. Lane 2: 10-fold dilution of the purified scFv. Lanes 3–8: two-fold serial dilutions of standard BSA from 2 mg/mL to 0.0625 mg/mL.

The sequences of the variable regions of E10E-1-10 were linked with a (G_4_S)_3_ linker to form a functional scFv. The DNA sequence corresponding to the scFv was subcloned into the vector, pcDNA3.1-V5/His, and transfected to Expi293F™ cells for transient expression, and the expression was confirmed by using the Western blotting probed by the anti-V5 tag antibody (Figure 2A). A high level of expression of the scFv was observed in Expi293F™ cells. Therefore, the expression of recombinant scFv was scaled up for purification by IMAC followed by SEC. The purity of the scFv was verified by SDS-PAGE and Coomassie blue staining (Figure 2C). The SEC analysis indicated some aggregation formed by the recombinant scFv (Figure 2B). The fractions corresponding to the main elution peak of scFv were collected and concentrated for the following experiments.

### 3.3. Binding Ability Estimation of scFv toward Homogenous and Heterogeneous PEDV Virions by Immunostainings and ELISA

**Figure 3:**
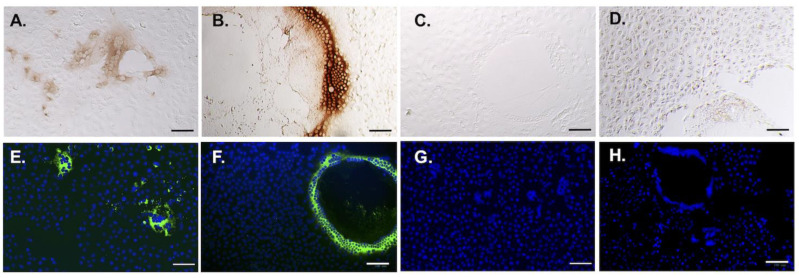
ICC and IFA of scFv against PEDV-infected cells. The purified scFv was used as the primary antibody to stain PEDV-PT (G2 PEDV) and CV777(G1 PEDV). The DAB coloration substrate was added for visualization in ICC (**A**–**D**); DAPI was used to stain the nuclei in IFA (**E**–**H**). (**A**,**E**) The immunostainings of scFv against PEDV-PT-infected cells. (**B**,**F**). The immunostainings of scFv against CV777-infected cells. (**C**,**G**). The controls of immunostainings of PEDV-PT-infected cells. (**D**,**H**) The controls of immunostainings of CV777-infected cells. The bars represented 100 μm in length.

The recombinant scFv was used for the immunocytochemical staining (ICC) and immunofluorescence assay (IFA) to verify its binding to PEDV virions. Both cells infected with G1 PEDV (CV777, the historic vaccine strain) and G2b PEDV (PEDV-PT, the parental strain of the scFv) were used to test the reactivity of the scFv. As expected, the recombinant scFv was able to probe the PEDV-infected cells with an obvious cytopathic effect as well as the peripherally scattered infected cells (Figure 3). Despite the same titers used to infect the cells, however, the morphologies of the cytopathic effects conferred by the two different PEDV strains were very different. The parental strain, PEDV-PT, formed small and scattered fusion cells, whereas CV777 formed giant fusion cells. Regardless of the cellular morphologies, the scFv was capable of recognizing both G1 and G2b viruses. The binding ability of scFv against homogenous and heterogeneous PEDV was estimated by ELISA. Both PEDV-PT and CV777 virions were coated on the plates and incubated with different amounts of the scFv. The parental IgG of scFv, E10E-1-10, was used as a positive control to calculate the sample-to-positive ratio (S/P ratio), which is defined as:

$$S/P\;\mathrm{ratio}=\frac{{\mathrm{OD}}_{\mathrm{sample}}-{\mathrm{OD}}_{\mathrm{negative}\;\mathrm{control}}}{{\mathrm{OD}}_{\mathrm{positive}\;\mathrm{control}}-{\mathrm{OD}}_{\mathrm{negative}\;\mathrm{control}}}$$

**Figure 4:**
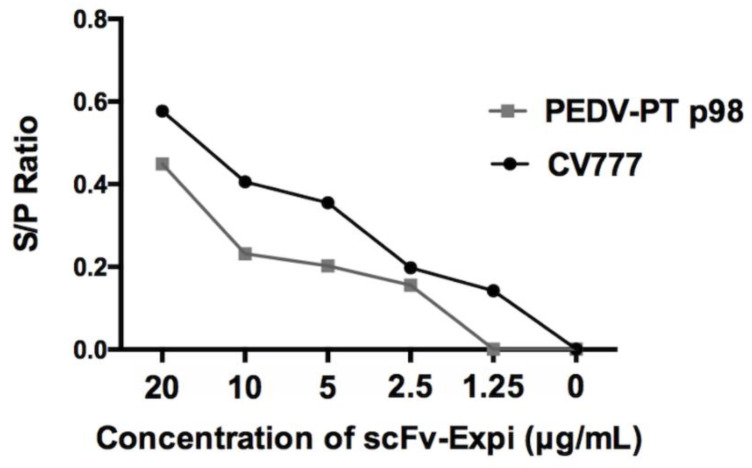
Evaluation of scFv binding toward PEDV virions by ELISA. The virions of PEDV-PT (G2 PEDV) and CV777 (G1 PEDV) were coated as the antigens on plates and probed with the serially diluted scFv. The S/P ratio is plotted as a function of the scFv concentration.

The S/P ratio for both viruses positively correlated with the concentration of scFv. Furthermore, our scFv can cross-react to both virus strains, indicating the cross-reactivity of this recombinant scFv (Figure 4).

### 3.4. Binding Ability of scFv with Purified PEDV S Protein by Using Pull-Down Assay and SEC 

**Figure 5:**
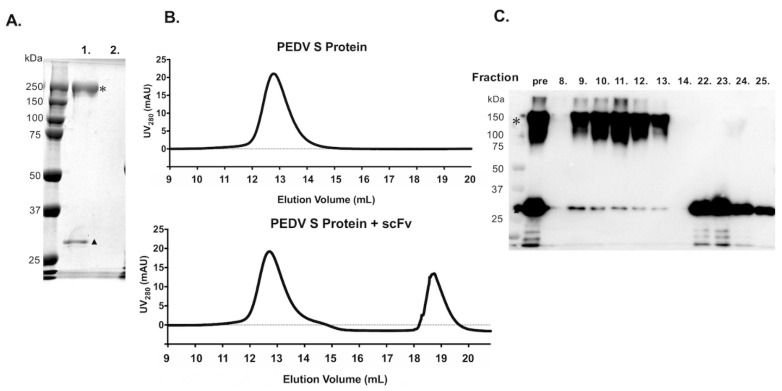
Evaluation of binding between scFv and PEDV S protein by MWCO filtration and SEC. (**A**) The SDS-PAGE of 100 kDa MWCO filtrated scFv with (lane 1) and without (lane 2) the PEDV S protein. (**B**) Size-exclusion chromatograms of the PEDV S protein without (top) and with (bottom) the scFv. (**C**). Western blot analysis of the elution fractions corresponding to the two elution peaks of the scFv mixed with the PEDV S protein (bottom chromatogram of (**B**)), sorted by SEC. The expected bands of PEDV S protein were labeled with asterisks. The retained scFv after the 100 kDa MWCO filtration was labeled with sloid triangle.

To further confirm that the PEDV S protein contains the epitope recognized by the scFv, an immunoprecipitation combined pull-down assay and SEC analysis were performed. For the immunoprecipitation assay, the purified trimeric PEDV S glycoprotein, which harbored a V5 tag and a His_x6_ tag, was incubated with scFv for 3 h and size-filtrated by centrifugation with a 100 kDa molecular weight cut-off (MWCO) spin column. In the absence of the PEDV S protein, all scFv passed through the 100 kDa MWCO spin column in the control group, and no protein band was detected in the respective lane of the SDS-PAGE (lane 2 in Figure 5A). The addition of the PEDV S protein indeed retained the scFv after the 100 kDa MWCO filtration (lane 1 in Figure 5A). SEC analysis of the PEDV S protein with excess scFv showed a similar elution volume as that without the scFv. The lack of an apparent shift in the elution volume could potentially be due to the relatively small change in the molecular size of the PEDV S protein when bound to the scFv (Figure 5B). To ascertain that the scFv was indeed co-eluted with the PEDV S protein during the SEC, the elution fractions corresponding to the PEDV S protein were analyzed by Western blotting. It was clear that some scFv co-eluted with the PEDV S protein (fractions 8–13), albeit the limited quantities relative to the unbound scFv that eluted later (fractions 22–25; Figure 5C).

### 3.5. Neutralizing Test 

**Figure 6:**
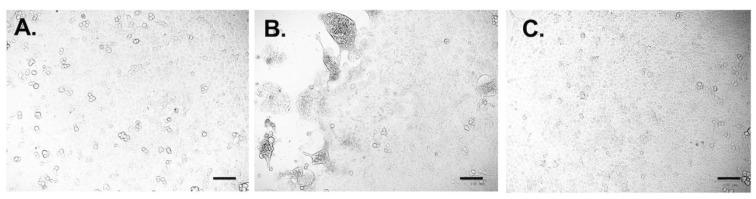
The observation of cytopathic effects (CPEs) in the neutralization test. The recombinant scFv was incubated with 400 TCID_50_/mL PEDV-PT-passage 5 for 2 h and subjected to the Vero cells. The CPE was observed every 24 h and the images were taken at the end time point, 72 h. (**A**) The group was supplemented with 25 μg/mL scFv and virus. (**B**) The challenge control group (virus without the scFv). (**C**) The non-challenged control group (no virus, no scFv). The black bars represented 100 μm in length.

The neutralizing ability of scFv against virulent G2b PEDV (PEDV-PT) and G1 PEDV CV777 was evaluated by performing a neutralization test and the end-point cell viability test. The scFv was able to completely block the infection and the development of CPE- and PEDV-PT-infected Vero cells at 24 hpi by using 6.25 μg/mL scFv, at 48 hpi by using 12.5 μg/mL scFv, and at 72 hpi by using 25 μg/mL scFv (Figure 6; Table 3). As shown in Figure 6, at 72 hpi, no obvious CPE was observed in the no-challenged control group and in the group supplemented with 25 μg/mL scFv compared with the severe CPEs in the challenged control group. No detectable viral neutralizing ability of the E10E-1-10 scFv against PEDV CV777 was noted (data not shown).

## 4. Discussion

Since 2010, the highly virulent G2 PEDV strain has significantly impacted the pork industry in several American and Asia countries [1]. With the high enteropathogenicity of the G2 PEDV strain, the suffering neonatal piglets die within a few days after infection [1]. As an enteric pathogen, the primary route to protect the neonates is to elicit passive lactogenic protection from sows [12,35]. However, stimulation of mucosal immunity by using a safe vaccine remains challenging, not least because of coronaviruses’ high heterogeneity and high mutation potential [36]. Considering the commercial importance of the global pork industry, an effective therapeutic strategy that could cut down the losses of pig numbers due to PEDV outbreaks is urgently needed. 

In this study, we established the repertoire of the VL chain and VH chain of the anti-PEDV S1^A^ neutralizing monoclonal antibody, E10E-1-10, based on which an engineered scFv was constructed. The selectivity of the scFv for the parental virus, namely PEDV-PT (G2 PEDV), and the heterologous virus, CV777 (G1 PEDV), were confirmed by the ICC, IFA, virion-based ELISAs, and SEC. While the scFv was capable of recognizing both G1 and G2b PEDV S proteins and neutralizing the homologous G2b PEDV, the scFv was unable to neutralize the heterologous G1 PEDV CV777. Indeed, effective antigen-binding does not necessarily warrant efficient neutralization against the target virus. Nevertheless, this PEDV-specific neutralizing scFv has the potential to be further developed into a therapeutic against homologous PEDV.

We previously reported that E10E-1-10 recognizes amino acids 435–485 of the G2b PEDV S protein [18]. Despite the two substitutions (V^441^I and S^477^A) within the G2b PEDV-PT S protein compared to the classical vaccine strain of G1 PEDV, CV777, our scFv could recognize both viruses, but it failed to neutralize the heterologous G1 PEDV CV777 [7]. Our result suggests that the V^441^I and S^477^A substitutions of the PEDV S protein may be critical for the binding strength between the paratope and epitope, therefore contributing to the different outcomes of virus neutralization. Interestingly, the V^441^I and S^477^A substitutions identified in the historic PEDV CV777 strain were not identified in the recently emerged Australian and European G1 PEDV strains, such as PEDV-25-10-2015-AUT (Australia, GenBank no. KT206204.1), PEDV-OH851 (USA, GenBank no. KJ399978), PEDV-UU (Netherland, GenBank no. KU985229.1), PEDV 1842/2016 (Italy, GenBank no. KY111278.1), and PEDV-GER/L00719/2014 (Germany, GenBank no. LM645058.1). The cross-reactivity and the neutralizing ability of the scFv against novel G1 PEDV strains and other G2 PEDV stains may be further characterized by constructing the strain-specific infectious clones [37]. The mechanisms and the critical determinant of the cross-reactivity and neutralizing ability of the S1^A^-specific scFv may be further studied and characterized by comparing the ultrastructural differences of binding patterns between G1 and G2 PEDV strains.

In veterinary medicine, therapeutic mAbs have been developed for years, especially in the treatment of cancer and chronic inflammation in companion animals. Nevertheless, licensed therapeutic mAbs remain rare [38]. The caninized anti-IL31 Ab, also known as lokivetmab, has been granted a conditional license for use in improving canine atopic dermatitis [39]. Although the officially approved therapeutic Ab is rare in veterinary medicine, several mAbs are now in clinical trials for therapeutic purposes in response to the increasing market [38]. Along with the anti-cancer and anti-inflammation mAb, therapeutic mAbs have been developed and applied in treating several infectious diseases. The scFv and single-domain antibody (sdAb) against *Staphylococcus aureus* were designed and utilized to control bovine mastitis [40,41]; the use of mouse-pig chimeric antibody against *Haemophilus parasuis* showed increasing survival rates *in vivo* [42]; the anti-foot and mouth disease (FMD) sdAb was able to reduce the viremia and viral shedding in pigs after challenge [43]. 

The route of administrating therapeutic mAbs depends on the infection routes of the diseases of interest and the pharmacokinetics of the mAb. Based on our understanding of mucosal immunity and host defense systems, oral administration is the most direct way to block the infection of enteric antigen [12,36]. Although there are challenges associated with oral delivery of proteinous therapeutics due to the low gastric pH, high proteolytic activity caused by gastric enzymes, bacterial metabolism, and other factors, successful oral administration of several therapeutic antibodies has been documented [44]. For instance, the administration of anti-F5 fimbriae scFv is used prophylactically for controlling neonatal calf colibacillosis [45]; a reduction of oocysts in the feces of chickens fed with anti-Eimeria scFv-expressing plants was observed [46]; and the oral administration of an scFv against PEDV was also developed and significantly increased the survival rates and resolved the clinical signs in neonatal piglets [37]. 

In the future, the scFv described herein will be evaluated for the following: proteomic stability in a harsh environment mimicking the digestive tract, the effective dosage in neonatal pigs, the practical formula, and the efficacy of oral treatments in vivo, to evaluate its effectiveness in mitigating urgent pig losses during outbreaks in farms. To achieve this goal, further optimizations for improving the stability of scFv in the digestive tract, such as compacting with mucoadhesive polymers, packing into emulsions or nanoparticles, surface displayed by the probiotics, and applying with enzymatic inhibitors [47]. Based on the neutralizing antibody we had, it is worth establishing a method for developing oral therapeutic antibodies in pigs and referring to other economic animals.

## 5. Conclusions

The anti-PEDV S1^A^ NmAb-derived scFv was successfully constructed and expressed in milligram quantity by a mammalian protein expression system. This recombinant scFv was capable of probing the virus-infected cells as well as the virions of both G1 and G2 PEDVs by recognizing the PEDV S. The scFv had cross-reactivity and showed great binding ability toward both parental G2b PEDV-PT, a PEDV strain with high virulence, and G1 PEDV-CV777, a highly passaged vaccine strain with low virulence. This scFv only showed neutralizing ability in vitro against the parental G2b PEDV-PT but not the G1 PEDV-CV777. The breadth of the neutralizing ability of the scFv should be further characterized. Based on the neutralizing pattern, the anti-PEDV S1^A^ scFv presented herein may serve as a potential therapeutic candidate against the virulent G2b PEDV in the field.

## Figures and Tables

**Table 1:** The primers and the general template-switch oligonucleotide (TSO) used in the primer-specific reverse transcription of mRNA.

Primer Name	Primer Sequence
Template-switch oligonucleotide (TSO)	5′-AAGCAGTGGTATCAACGCAGAGTACATGrGRrGr *-3′
mIgK RT	5′-TTGTCGTTCACTGCCATCAATC-3′
mIgL RT	5′-GGGGTACCATCTACCTTCCAG-3′
mIgHG RT	5′-AGCTGGGAAGGTGTGCACAC-3′

*: Three riboguanosines (rGrGrG) were added at the 3′ end of the TSO.

**Table 2:** The primers used in the PCR reactions. Along with the common forward primer (ISPCR), the primers for variable kappa (mIgKpcr), variable lambda (mIgLpcr) light chains, and variable heavy chain (mIgHGpcr) were utilized to amplify specific sequences.

Primer Name	Primer Sequence
ISPCR	5′-AAGCAGTGGTATCAACGCAGAG-3′
mIgKpcr	5′-ACATTGATGTCTTTGGGGTAGAAG-3′
mIgLpcr	5′-ATCGTACACACCAGTGTGGC-3′
mIgHGpcr	5′-GGGATCCAGAGTTCCAGGTC-3′

**Table 3:** The neutralizing ability of recombinant scFv against the G2b PEDV.

Time Post-Challenge (hr)	Concentration of scFv (µg/mL)	Protection
24	6.25	No CPE
	12.5	No CPE
	25	No CPE
48	6.25	Few CPE
	12.5	No CPE
	25	No CPE
72	6.25	CPE
	12.5	Few CPE
	25	No CPE
